# Oxidative Stress Indicated by Nuclear Transcription Factor Nrf2 and Glutathione Status in the Blood of Young Children with Autism Spectrum Disorder: Pilot Study

**DOI:** 10.3390/antiox14030320

**Published:** 2025-03-06

**Authors:** Magdalena Chełchowska, Joanna Gajewska, Elżbieta Szczepanik, Joanna Mazur, Agnieszka Cychol, Aleksandra Kuźniar-Pałka, Jadwiga Ambroszkiewicz

**Affiliations:** 1 Department of Screening Tests and Metabolic Diagnostics, Institute of Mother and Child, Kasprzaka 17a, 01-211 Warsaw, Poland; joanna.gajewska@imid.med.pl (J.G.); agnieszka.cychol@imid.med.pl (A.C.); jadwiga.ambroszkiewicz@imid.med.pl (J.A.); 2 Clinic of Paediatric Neurology, Institute of Mother and Child, Kasprzaka 17a, 01-211 Warsaw, Poland; elzbieta.szczepanik@imid.med.pl (E.S.); aleksandra.kuzniar@imid.med.pl (A.K.-P.); 3 Department of Humanization in Medicine and Sexology, Collegium Medicum, University of Zielona Góra, 65-729 Zielona Góra, Poland; j.mazur@cm.uz.zgora.pl

**Keywords:** autism spectrum disorders, nuclear factor erythroid 2-related factor 2, glutathione, glutathione peroxidase 3, glutathione reductase, oxidative stress, antioxidants

## Abstract

This pilot study investigated the relationship between nuclear transcription factor Nrf2 and glutathione homeostasis in children with autism spectrum disorder (ASD), addressing the role of oxidative stress in ASD pathophysiology. Oxidative stress, characterized by an imbalance between reactive oxygen species and antioxidant defenses, has been implicated in ASD and may contribute to neuroinflammation and mitochondrial dysfunction. Nrf2, a key regulator of the antioxidant response, influences glutathione synthesis and recycling, making it critical for cellular redox balance. This study included 23 children with ASD and 21 neurotypical healthy controls, and measured levels of Nrf2, Keap1 (Kelch-like ECH-associated protein 1), reduced glutathione (GSH), oxidized glutathione (GSSG), glutathione reductase (GR), and peroxidase (GPx3) in blood samples. Our study reveals altered antioxidant defense in children with autism spectrum disorder, as evidenced by reduced levels of Nrf2, Keap1, GSH, and GR, along with elevated GSSG and a lower GSH/GSSG ratio. These findings indicate an increased oxidative stress burden in this population. Additionally, the observed positive correlation between Nrf2, GSH, and GR levels suggests an important role for Nrf2 in maintaining glutathione homeostasis. Our results underscore the potential involvement of oxidative stress in ASD and emphasize the need for further research into targeted therapeutic approaches to address this imbalance.

## 1. Introduction

Autism spectrum disorders (ASDs) are complex neurodevelopmental conditions that manifest in early childhood and are characterized by deficits in communication and social skills as well as repetitive and stereotypical behaviors [1]. There is significant variability among individuals, with each person presenting a unique combination of symptoms and associated health challenges. The multifactorial nature of ASD, involving genetic, environmental, immunological, and neurological factors, makes it difficult to identify a single cause or universal treatment approach. A growing body of evidence highlights the role of oxidative stress in the pathophysiology of ASD, adding another layer of complexity to our understanding of this disorder [2,3].

Oxidative stress refers to an imbalance in the production of reactive oxygen species (ROS) or reactive nitrogen species (RNS) and the ability of the body to neutralize them with antioxidants. This imbalance can lead to cellular damage that affects various biological systems, including the brain. In people with ASD, oxidative stress is thought to contribute to neuroinflammation, which can exacerbate core symptoms such as communication deficits, social interaction difficulties, and repetitive behaviors [4].

The relationship between oxidative stress, mitochondrial dysfunction, and immune dysregulation is particularly relevant in ASD [5,6,7,8]. Mitochondria, the energy producers in cells, are highly susceptible to oxidative damage. Emerging evidence suggests that mitochondrial dysfunction is prevalent in children with ASD, potentially affecting energy metabolism, which is critical for brain development and function. Similarly, immune dysregulation—whether overactive or underactive—can increase inflammation and oxidative stress, leading to the systemic problems often observed in ASD, such as gastrointestinal problems, sleep disturbances, and neurological disorders [9,10,11].

Nuclear factor erythroid 2-related factor 2 (Nrf2) is a key transcription factor that regulates the expression of more than 500 genes involved in cellular defense against oxidative stress, particularly those linked to antioxidant pathways. Nrf2 typically binds to Kelch-like ECH-associated protein 1 (Keap1) and remains sequestered in the cytoplasm under non-stress conditions. Keap1 acts as a sensor of oxidative stress through its reactive cysteine residues, which are highly sensitive to ROS and RNS. When oxidative stress levels rise, specific cysteine residues in Keap1 are oxidized, causing a conformational change that releases Nrf2 from the Keap1-Nrf2 complex. Once free, Nrf2 translocates to the nucleus, where it binds to the antioxidant response element (ARE) in the promoter regions of target genes, leading to the transcription of several antioxidant and cytoprotective genes, many of which are involved in glutathione (GSH) metabolism [12,13].

GSH, or γ-glutamylcysteinylglycine, is the primary endogenous non-enzymatic antioxidant and the most abundant thiol in the central nervous system. It plays a vital role in various cellular functions, including transcription, replication, cell growth, enzyme regulation, immune defense, and apoptosis. In the brain, GSH functions as a neurotransmitter, modulates glutamatergic transmission, and protects neurons by chelating metal ions and preventing harmful Fenton reactions. The primary antioxidant function of glutathione involves reducing hydrogen peroxide to water and oxygen and converting organic hydroperoxides into alcohols. During this process, glutathione peroxidase (GPx) oxidizes reduced glutathione (GSH) to glutathione disulfide (GSSG), which is then restored to its reduced form by glutathione reductase (GR). This enzymatic cycle maintains the crucial GSH/GSSG balance for cellular function and survival [6,14,15,16].

In addition to GSH regeneration from GSSG, glutathione levels are also increased by neosynthesis driven by glutamate-cysteine ligase (GCL). Activated by Nrf2, GCL catalyzes the rate-limiting step in glutathione synthesis by combining glutamate and cysteine, a key sulfur-containing amino acid required for GSH production. Nrf2 further upregulates glutathione S-transferase (GST), an enzyme that uses GSH to detoxify harmful substances, and NADPH oxidoreductase, which maintains the NADPH pool—an essential cofactor for glutathione reductase [17,18].

By upregulating enzymes involved in both the synthesis and recycling of glutathione, Nrf2 plays a critical role in maintaining cellular redox balance. By promoting GSH production and recycling, the Nrf2 pathway enhances the ability of the cell to buffer oxidative stress by neutralizing free radicals and preventing oxidative damage to proteins, lipids, and DNA. This coordinated response between Nrf2 signaling and the GSH/GSSG system ensures that cells remain protected from oxidative damage and maintain homeostasis [17,19]. Studies have demonstrated that children with autism have lower levels of reduced glutathione (GSH), higher levels of oxidized glutathione (GSSG), reduced total glutathione (tGSH), and a decreased GSH/GSSG ratio in plasma and brain regions such as the cerebellum and temporal cortex compared to neurotypical children [7,14,20,21,22,23,24,25]. However, data on Nrf2 levels and Nrf2/Keap1 activity in blood cells from individuals with ASD remain limited and inconsistent in some cases. Findings have revealed both reduced Nrf2 protein expression and activity in the frontal cortex and monocytes, as well as alterations in Nrf2 mRNA levels in peripheral blood mononuclear cells (PBMCs) from individuals with ASD [26,27,28]. Only one study has compared Nrf2 and Keap1 levels in the serum of children with ASD and healthy controls, focusing on their impact on heme oxygenase-1 (HO-1) expression [29]. In addition, there is a lack of research on the relationship between glutathione status and Nrf2 levels in the blood of children with autism spectrum disorders. The aim of this study was to measure the levels of Nrf2, Keap1 protein, glutathione, and key enzymes involved in its metabolism in the blood of young patients in both autism and neurotypical groups. In addition, this study was an attempt to evaluate the relationship between Nrf2 and markers of glutathione status in both groups.

## 2. Materials and Methods

### 2.1. Participants

This study was conducted at the Institute of Mother and Child in Warsaw and included patients from the Clinic of Pediatric Neurology between May 2022 and November 2024. A total of 23 children with ASD (7 girls and 16 boys), aged between 1.6 and 4 years, were included, along with a control group of 21 neurotypical healthy children (8 girls and 13 boys) of the same age. All participants were Caucasian. The diagnosis of ASD was confirmed based on the 10th revision of the International Classification of Diseases (ICD-10) by pediatric psychiatrists, supported by psychological examination and observation [30]. Children with ASD underwent neurological observation and differential diagnosis in the pediatric neurology unit to avoid misdiagnosis.

The inclusion criteria for the study group were a confirmed diagnosis of ASD, normal neurological findings, and a history of uncomplicated pregnancy and delivery. Exclusion criteria included any chronic illness requiring long-term treatment; significant hearing or visual impairment; current antibiotic therapy; or the use of anti-inflammatory, anti-allergy, antioxidant, or immunomodulatory drugs/supplements. Additional exclusion criteria were based on the presence of an elimination diet or acute or chronic infection within one month prior to blood collection. The control group had the same inclusion and exclusion criteria, except for the diagnosis of autism spectrum disorder.

This study was carried out in accordance with the ethical standards of the Declaration of Helsinki after approval by the Ethics Committee of the Institute of Mother and Child (decision number 16/2022, date of approval 5 May 2022). Parents of all participants were informed of the aims and procedures of this study and provided written consent for the analysis of biological samples and use of clinical information from the patients’ medical records.

### 2.2. Blood Processing

For biochemical assessments, 4 mL of venous blood was collected into EDTA-containing vacutainer tubes from each participant early in the morning after an overnight fast. Blood samples were processed according to the specified requirements for biochemical analysis. After centrifugation, the resulting plasma was divided into small portions and immediately stored at −80 °C for future analysis.

### 2.3. Biochemical Analysis

Plasma levels of antioxidant defense markers were measured using precise immunoenzymatic assays, strictly following the manufacturer’s guidelines at every step.

The concentrations of nuclear factor erythroid-derived 2-like 2 (NFE2L2 or Nrf2) and Keap1 were measured using ELISA kits (Human NFE2L2 ELISA Kit Cat. No.: EH3417 and Human KEAP1 ELISA Kit Cat. No.: EH4240, Fine Biotech Co., Ltd., Wuhan, China) based on sandwich ELISA technology. Both assays followed a similar procedure: Monoclonal antibodies specific to NFE2L2 or Keap1 were pre-coated onto 96-well plates. Samples and standards were added to the wells, and after incubation, unbound substances were washed away. Biotinylated detection antibodies specific to NFE2L2 or Keap1 were then added. After removing the excess detection antibodies, horseradish peroxide Streptavidin conjugates were added. The colorimetric reaction was initiated by adding TMB (3,3’,5,5’-tetramethylbenzidine), which reacted with HRP to produce a blue color. The reaction was stopped by adding a stop solution, turning the color from blue to yellow. The intensity of the yellow color, measured at 450 nm using a microplate reader, was directly proportional to the concentration of the target protein in the samples. The concentrations of NFE2L2 and Keap1 were calculated by comparing the optical density (OD) values at 450 nm with a standard curve. The intra- and inter-assay coefficients of variation for NFE2L2 and Keap1 were less than 8.0% and 10%, respectively, with detection sensitivities below 0.094 ng/mL for NFE2L2 and 14.063 pg/mL for Keap1.

GSH and GSSG levels were quantified using sandwich enzyme-linked immunosorbent assay (ELISA) kits using two highly specific monoclonal antibodies (Human GSH ELISA Kit, Cat. No.: 201-12-5407; Human GSSG ELISA Kit, Cat. No. 201-12-5444, SunRed Bio-Technology Company, Shanghai, China). The cellular redox index was assessed by calculating the GSH/GSSG ratio (R).

The levels of glutathione reductase (GR) and glutathione peroxidase 3 (GPx3) were similarly determined using appropriate ELISA kits (Human Glutathione Reductase ELISA Kit, Cat. No.: MBS2703164, and Human Glutathione Peroxidase 3 ELISA Kit, Cat. No: MBS2881280, MyBioSource Inc., San Diego, CA, USA). The intra- and inter-assay coefficients of variation were less than 8.0% and 10% for GPx3 and 10% and 12% for GSH, GSSG, and GR. The detection sensitivities of the assays were less than 0.118 μmol/L for GSH, 0.045 μmol/L for GSSG, 0.230 ng/mL for GPx3, and 35.000 pg/mL for GR. Detailed descriptions of these methods can be found in our previous studies [31].

### 2.4. Statistical Analysis

Baseline characteristics were presented as means with standard deviation or medians with interquartile range, depending on whether the data were normally distributed. The correlation between the studied variables was assessed using Spearman’s coefficient. The distributions of the values of the analyzed parameters in the autistic children and control groups were compared using the Mann–Whitney (MW) test. The level of difference between groups was assessed by the r effect size measure, calculated as the standardized MW test (z) divided by the root of N, with 0.30, 0.50, and 0.80 interpreted as small, medium, and large effect sizes, respectively. In addition, a general linear model (GLM) was used, examining the main effect of age, sex, and group membership and the 2 -way interaction between gender or age and group as predictors of each parameter. Statistical Package for the Social Sciences (SPSS) Version 29.0 (IBM Corp, Armonk, NY, USA) was used, and *p* values <0.05 were considered statistically significant.

## 3. Results

The mean age of the children in the two study groups did not differ significantly (*p* = 0.671). The average age in the autism group was 3.2 years, while in the control group it was 2.9 years. There were also no sex differences between the groups (*p* = 0.592).

The differences in the plasma concentrations of the antioxidant parameters between the two groups are presented in Table 1.

Children with autism had significantly lower plasma levels of GSH, GR, Nrf2, and Keap1 (*p* < 0.05), as well as a lower R-index value (*p* < 0.001), compared to the healthy children. Conversely, GSSG concentrations were significantly higher in the autism group (*p* < 0.01), whereas GPx3 levels were not significantly different between the groups.

**Figure 1:**
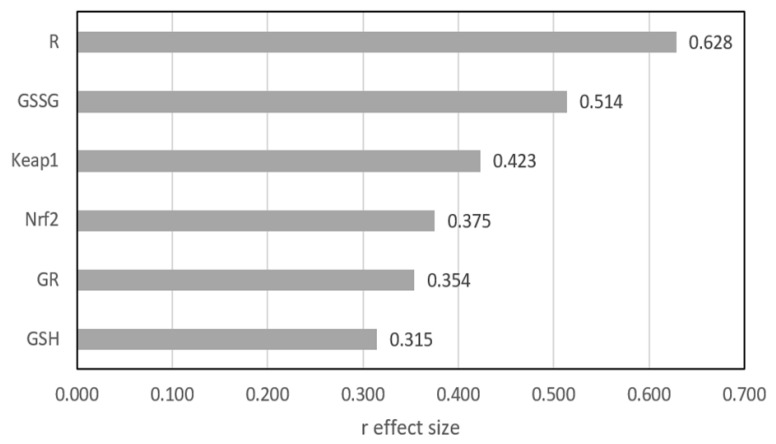
Mann–Whitney r effect size comparison of two groups. GSH—reduced glutathione; GSSG—oxidized glutathione; GPx3—glutathione peroxidase; GR—glutathione reductase, R (GSH/GSSG ratio)—index of the cell’s redox; Nrf2—nuclear factor erythroid-derived 2-like protein 2; Keap1—Kelch-like ECH-associated protein A.

Figure 1 shows the differences between the two groups as expressed by the r effect size measure, except GPx3, for which no statistically significant difference was found. The largest difference was shown for R and GSSG, where the r value exceeded 0.5, indicating a medium effect. For the other four parameters, the differences were significant, but the effect size qualified them at a small level.

It was confirmed that neither sex nor age had an impact on the inference of differences between the groups. The results of the GLM estimation indicate a statistically significant difference between the study and control groups for all parameters except GPx3. None of the seven models showed a significant main effect of sex or age on the variability of the respective parameter, nor a significant two-way interaction between sex or age and group.

Table 2 presents a detailed analysis of the correlations between selected antioxidant parameters for the entire group of children, as well as for each group separately. In the whole group, plasma GSH concentrations positively correlated with GR, R-index, Nrf2, and Keap1 levels. Similarly, Nrf2 levels were significantly associated with Keap1, GR, and R-index. A positive correlation was also found between GR and GPx3 levels. Conversely, GSSG levels were negatively correlated with the R-index.

**Figure 2:**
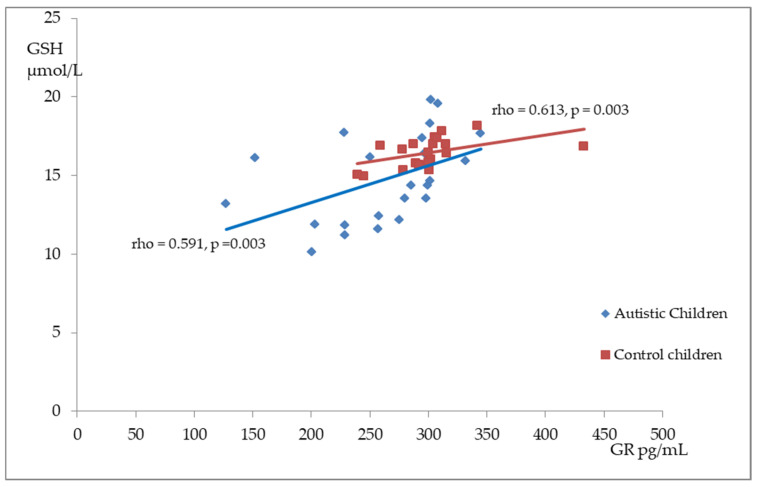
A scatterplot illustrating the correlation between reduced glutathione (GSH) and glutathione reductase (GR) levels in children with autism compared to the control group.

**Figure 3:**
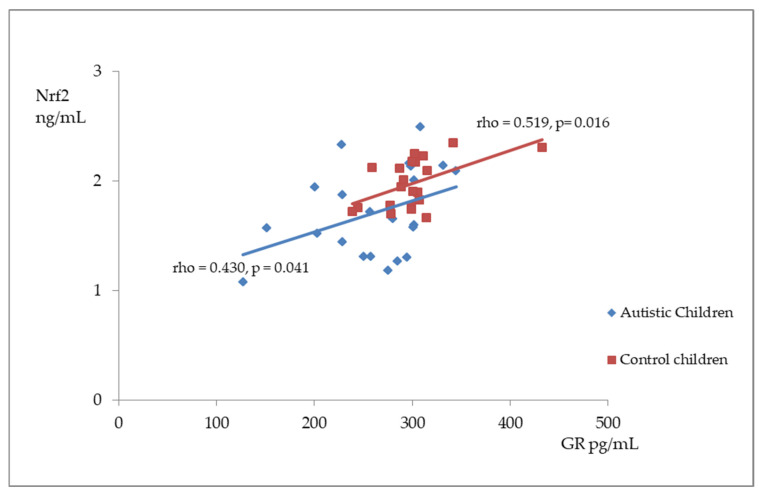
A scatterplot illustrating the correlation between nuclear factor erythroid-derived 2-like 2 (Nrf2) and glutathione reductase (GR) levels in children with autism compared to the control group.

**Figure 4:**
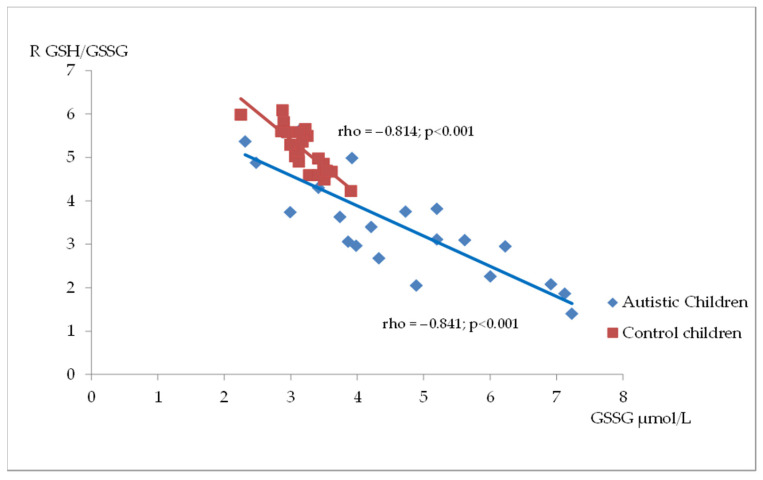
A scatterplot illustrating the correlation between oxidized glutathione (GSSG) and GSH/GSSG ratio (R) levels in children with autism compared to the control group.

Figure 2, Figure 3 and Figure 4 illustrate the significant associations between the studied parameters in both the control and ASD groups. In both groups, GR levels demonstrated a positive correlation with GSH (Figure 2) and Nrf2 concentrations (Figure 3). Moreover, a negative correlation was observed between GSSG levels and the R-index (Figure 4).

Additionally, in the ASD group, a positive correlation was observed between GR and GPx3 levels (*p* < 0.01). Meanwhile, in the control group, GR levels were positively correlated with R-index values (*p* < 0.01). No other correlations were confirmed between antioxidant defense parameters in the two groups.

## 4. Discussion

Oxidative stress in ASD is characterized by reduced activity of antioxidant enzymes (superoxide dismutase, SOD, catalase, CAT, and GPx) and increased markers of cellular damage, such as DNA oxidation (8-hydroxy-2’-deoxyguanosine, 8OHdG), lipid peroxidation (malondialdehyde, MDA, and thiobarbituric acid-reactive substances, TBARSs), and protein oxidation (carbonyl proteins) [2,32,33,34,35,36]. Important biomarkers of oxidative stress in ASD are advanced glycation end products (AGEs). The observed accumulation of plasma protein glycation and oxidation markers in ASD may be significant in predicting disease progression [37,38].

The results of our study indicated reduced Nrf2 levels, accompanied by diminished reduced glutathione and an altered GSH/GSSG ratio, confirming the presence and severity of oxidative stress in young children with autism. Additionally, the observed positive correlation between Nrf2 levels and GSSG and GR concentrations in the entire study group suggests significant relationships between these parameters.

Reduced Nrf2 expression has been linked to several brain disorders, including Parkinson’s disease, amyotrophic lateral sclerosis, and Alzheimer’s disease, and its function appears to be weakened in depression [39,40,41,42]. Evidence suggests that Nrf2 levels and Nrf2/Keap1 activity may also be disrupted in autism spectrum disorders [30,31]. In our study, we found significantly lower levels of Nrf2 and Keap1 proteins in the plasma of autistic children than in neurotypical healthy children. These results are consistent with an in vitro study by Nadeem et al. [26,43], who examined blood cells from autistic children. Their findings showed that children with ASD have reduced Nrf2 protein expression and nuclear binding activity in monocytes and neutrophils compared with typically developing children. This decrease could be associated with increased inflammation and nitrative stress, potentially characterized by higher levels of pro-inflammatory cytokines (IL-6 and IL-1β) and impaired antioxidant responses to environmental stressors, which may contribute to the oxidative imbalance observed in ASD [26,43]. On the other hand, a more recent study by Bolotta et al. [28] found a modest but significant increase in Nrf2 mRNA levels in PBMCs from individuals with ASD. This raises the possibility that, despite elevated mRNA levels, there may be a defect in Nrf2′s ability to translocate to the nucleus. Nrf2 expression and activity are also downregulated in the frontal cortex, a region that plays a crucial role in higher cognitive functions such as decision-making, problem-solving, planning, attention, and the regulation of emotions and behavior [27]. In contrast, one study found elevated serum levels of Keap1 and Nrf2 in children with autism along with reduced levels of heme oxygenase-1 (HO-1), a key antioxidant enzyme whose gene expression is regulated by Nrf2 [29]. These discrepancies may result from the complex regulation of the Nrf2/Keap1 pathway in response to oxidative stress. Reduced Nrf2 and Keap1 activity or levels in autistic children may indicate protein depletion after excessive oxidative stress, while elevated levels could reflect an earlier phase of the response, where these proteins are upregulated to counteract oxidative damage.

As previously mentioned, malfunction of the Nrf2/Keap1 pathway, which controls Nrf2 stability and activation in response to oxidative stress, may further contribute to impaired antioxidant defenses, including the maintenance of adequate glutathione levels [17,18].

The mitochondrial abnormalities and inflammation found in autism are sources of elevated hydrogen peroxide levels, potentially leading to GSH dysregulation. Similar to other authors, we found lower levels of reduced glutathione and the GSH/GSSG redox index and elevated concentrations of oxidized glutathione in the plasma of children with ASD compared with healthy children [22,32,44,45]. The effect size measure used in our analyses, which ranks parameters according to the magnitude of differences between groups, proved particularly useful given the small sample size. It demonstrated that the effect size for the R-index and oxidized glutathione was notably larger than for the other parameters. Additionally, similar to others, we observed a strong negative association between these two parameters, indicating intracellular oxidative stress in children with ASD [22].

To the best of our knowledge, the positive correlation we observed for the first time between Nrf2 levels and both GSH concentrations and the GSH/GSSG ratio in the plasma of the studied children may confirm the role of this factor in maintaining glutathione homeostasis.

In contrast, we found no differences in GPx levels between the groups, likely because we measured the extracellular form of glutathione peroxidase (GPx3), which is primarily found in plasma, while others have assessed total GPx activity [32,36,46]. However, we demonstrated reduced GR activity in individuals with ASD, which contributed to the observed imbalance in the GSH/GSSG ratio in this group. As GR levels declined, we observed a corresponding reduction in reduced glutathione. Additionally, there was a positive correlation between GR and Nrf2 levels in both groups. The lower GR levels in the ASD group may result not only from reduced GR gene expression but also from a decreased expression of genes involved in nicotinamide adenine dinucleotide phosphate (NADPH) production and regeneration. Nrf2 regulates key enzymes of the pentose phosphate pathway, such as G6PD (glucose-6-phosphate dehydrogenase) and PGD (phosphogluconate dehydrogenase), which are the primary sources of NADPH in cells. By promoting NADPH production, Nrf2 supports antioxidant defenses and facilitates GSH regeneration from GSSG, enabling cells to manage oxidative stress better [13].

The decrease in GSH levels can also result from impaired de novo synthesis within the cell, which depends on the availability of key amino acids, such as cysteine and methionine, both of which are critical for glutathione production [47,48]. Cysteine plays a direct role in GSH synthesis, whereas methionine, through its involvement in the methionine cycle, can be converted into cysteine via the transsulfuration pathway. A recent meta-analysis reported significantly lower plasma concentrations of methionine and cysteine in individuals with ASD than in healthy controls [3]. Although we lacked specific data on the plasma levels of these amino acids in the children included in our study, this represents a limitation. However, the reduced level of plasma Nrf2 and Keap1 protein observed in our study may contribute to the altered expression of glutamate-cysteine ligase, the rate-limiting enzyme in de novo glutathione synthesis, further impacting GSH levels and cellular redox balance.

In humans, aging is associated with a decline in antioxidant defenses, as evidenced by reduced total GSH levels, lower GSH/GSSG ratios, and diminished activity of enzymes like GPx and GR, which contribute to increased oxidative stress and may play a role in the development of neurodegenerative diseases [49,50]. Studies have also shown a negative correlation between Nrf2 levels and age in children with ASD, particularly in those aged 3 to 12 years [29]. However, in our study, we did not observe an age-related impact on any of the examined parameters. This could be due to the fact that our research focused on a group of very young children, all within a narrow age range, which may limit the detection of age-related differences.

Also, sex differences in GSH metabolism have been observed in both animal models and humans [49,51,52,53]. In rats, females exhibited higher levels of hepatic mitochondrial GSH and increased GPx activity compared to males, but these levels decreased following ovariectomy [51]. However, our study did not find a significant sex-based effect on oxidative stress markers in young children with ASD, which contrasts with others studies suggesting that male mitochondria are more vulnerable to oxidative stress due to lower levels of reduced glutathione [52].

A limitation of our study is the small sample size, which may have reduced the significance of our findings. However, we conducted this study on a group of very young Caucasian children (average age: 3 years, range: 1.6–4.0 years) who had just been diagnosed with autism. As a result, these children had not undergone prior treatments, including antioxidant, anti-allergic, or immunomodulatory therapies. Since some oxidative stress parameters can be influenced by age and sex, the study and control groups were matched to avoid potential bias [49,50,51,52]. Despite the small sample size, this study serves as a valuable starting point for investigating oxidative stress within the broader context of metabolic research. This includes profiling amino acids, purines, pyrimidines, and organic acids, as well as considering allergological, genetic, and immunological factors. Our findings will help guide future research, providing insights into the interactions between oxidative stress and other metabolic disruptions in a larger group of young children with ASD.

## 5. Conclusions

In conclusion, our study provides evidence of impaired Nrf2 and glutathione homeostasis in children with autism spectrum disorder, highlighting a potential role of oxidative stress and ASD. Low Nrf2 levels, along with reduced glutathione and an altered GSH/GSSG ratio, may reflect an insufficient antioxidant response to oxidative stress in ASD. Furthermore, the decreased glutathione reductase activity and the significant correlation between Nrf2 and GR suggest that Nrf2 may regulate GR expression, contributing to the redox imbalance observed in children with autism spectrum disorder. These findings establish a basis for future research into the neuroimmunometabolic aspects of ASD and may offer valuable insights into potential therapeutic strategies.

## Figures and Tables

**Table 1:** Comparison of antioxidant defense markers between children with autism and healthy controls.

	Control Children *n* = 21	Autistic Children *n* = 23	*p-*Value
^a^ GSH (µmol/L)	16.44 ± 0.91	14.78 ± 2.80	0.037
^a^ GSSG (µmol/L)	3.19 ± 0.35	4.52 ± 1.46	0.001
^b^ R (GSH/GSSG)	5.17 [4.68–5.59]	3.53 [2.68–4.53]	0.000
^a^ GPx3 (ng/mL)	18.00 ± 2.28	17.47 ± 4.62	0.366
^b^ GR (pg/mL)	300.52 [282.83–309.45]	280.11 [228.73–301.23]	0.019
^a^ Nrf2 (ng/mL)	1.98 ± 0.22	1.72 ± 0.39	0.013
^a^ Keap1(pg/mL)	171.48 ± 25.09	149.08 ± 31.98	0.005

^a^ mean values and standard deviation (SD); ^b^ median values and interquartile range (1-3IQR); GSH—reduced glutathione; GSSG—oxidized glutathione; GPx3—glutathione peroxidase; GR—glutathione reductase, R (GSH/GSSG ratio)—index of the cell’s redox; Nrf2—nuclear factor erythroid-derived 2-like protein 2; Keap1—Kelch-like ECH-associated protein A.

**Table 2:** Spearman’s rank correlation coefficients between antioxidant defense markers in the entire group of studied children (*n* = 44), the control group (*n* = 21), and the ASD group (*n* = 23).

	Entire Group *n* = 44		Control Group *n* = 21		ASD Group *n* = 23	
	*rho*	*p*-Value	*rho*	*p*-Value	*rho*	*p*-Value
GSH/GSSG	−0.014	0.929	0.158	0.493	0.150	0.494
GSH/R	0.429	0.004	0.340	0.132	0.333	0.121
GSH/GR	0.632	0.000	0.613	0.003	0.591	0.003
GSH/GPx3	0.212	0.167	−0.377	0.092	−0.022	0.082
GSH/Nrf2	0.493	0.001	0.401	0.071	0.394	0.078
GSH/Keap1	0.453	0.002	0.377	0.096	0.277	0.200
GSSG/R	−0.855	0.000	−0.841	0.000	−0.814	0.000
GSSG/GR	−0.252	0.099	−0.368	0.101	−0.083	0.707
GSSG/GPx3	−0.095	0.540	0.109	0.638	−0.022	0.922
GSSG/Nrf2	−0.052	0.391	−0.052	0.823	0.024	0.914
GSSG/Keap1	−0.133	0.457	0.016	0.944	0.186	0.396
R/GR	0.512	0.000	0.593	0.005	0.329	0.125
R/Gpx3	0.162	0.292	−0.094	0.685	0.210	0.612
R/Nrf2	0.358	0.017	0.259	0.258	0.112	0.335
R/Keap1	0.253	0.097	0.199	0.388	−0.087	0.691
GR/GPx3	0.345	0.022	0.049	0.832	0.571	0.004
GR/Nrf2	0.530	0.000	0.465	0.034	0.430	0.041
GR/Keap1	0.305	0.044	0.432	0.050	0.029	0.897
Gpx3/Nrf2	0.071	0.071	0.060	0.797	0.009	0.968
Gpx3/Keap1	0.180	0.180	0.045	0.845	0.219	0.315
Nrf2/Keap1	0.408	0.006	0.319	0.066	0.175	0.733

GSH—reduced glutathione; GSSG—oxidized glutathione; GPx3—glutathione peroxidase; GR—glutathione reductase, R (GSH/GSSG ratio)—index of the cell’s redox; Nrf2—nuclear factor erythroid-derived 2-like protein 2; Keap1—Kelch-like ECH-associated protein A.

## Data Availability

All of the data is contained within the article.
